# Radial Endobronchial Ultrasound-guided Transbronchial Cryobiopsy *versus* Forceps Biopsy for the Diagnosis of Solitary Pulmonary Nodules: A Prospective Randomised Trial

**DOI:** 10.2174/0118743064262132230922110818

**Published:** 2023-09-22

**Authors:** Michael Brown, Phan Nguyen, Hubertus Jersmann, Mark Holmes, Michelle Wong

**Affiliations:** 1 Department of Thoracic Medicine, Royal Adelaide Hospital, Adelaide, Australia; 2 Faculty of Health and Medical Sciences, Adelaide Medical School, University of Adelaide, Adelaide, Australia; 3 Department of Respiratory and Sleep Medicine, Lyell McEwin Hospital, Adelaide, Australia

**Keywords:** Endobronchial ultrasound, Transbronchial cryobiopsy, Cryobiopsy, Solitary Pulmonary Nodule, Lung cancer, Peripheral pulmonary nodule

## Abstract

**Background:**

Improvements in pulmonary diagnostic imaging and the development of lung cancer screening are increasing the prevalence of Solitary pulmonary nodules (SPNs). Fluoroscopically guided radial endobronchial ultrasound (EBUS) with transbronchial forceps biopsy (TB-FB) has been the conventional diagnostic method. Transbronchial cryobiopsy (TB-CB) is an alternative biopsy method. We sought to compare transbronchial cryobiopsy to transbronchial forceps biopsy for the diagnosis of SPNs.

**Methods:**

A prospective, single-centre, randomised controlled trial was conducted at the Royal Adelaide Hospital (RAH). Patients with SPNs were randomised to either 5 transbronchial forceps biopsies or one transbronchial cryobiopsy. Complete blinding of investigators and participants was not possible, as transbronchial cryobiopsy required general anaesthesia. The primary outcome was diagnostic yield with secondary outcomes of specimen size, diagnostic yield for subsets challenging to access with forceps and safety.

**Results:**

The overall diagnostic yield for the 28 enrolled subjects was 76.8%(22/28). The diagnostic yield was 91.7% (11/12 patients) for transbronchial cryobiopsy and 68.8% (11/16 patients) for forceps biopsy (p=0.14). Median biopsy sizes were consistently larger for the cryobiopsy arm at 7.0mm compared to 2.5mm(p<0.0001). An eccentric EBUS image signalling the probe was adjacent to the nodule occurred in 4/28 cases, and TB-CB confirmed a diagnosis in 3/3 randomised to this arm. There were no major complications with either technique.

**Conclusion:**

Transbronchial cryobiopsy under the guidance of fluoroscopy and radial EBUS facilitates larger biopsy specimens without a significant increase in major complications. Further research is required to confirm the effect on diagnostic yield; however, our study supports a role for TB-CB in the diagnosis of SPNs and small, nodule-adjacent biopsies.

**Clinical Trial Registration Number:**

Reference number of R20160213(HREC/16/RAH/37).

## 1 INTRODUCTION

Solitary pulmonary nodules (SPNs) are focal parenchymal opacities completely surrounded by unaltered pulmonary parenchyma and cannot be visualised during endobronchial examination [1]. They are small (<30mm) in size and can be morphologically solid, part solid or ground glass [2]. The prevalence of pulmonary nodules in incidental finding studies is 2-24% and 17-53% in lung cancer screening populations [3]. Recent studies have demonstrated that lung cancer screening has a 20% reduction in lung cancer mortality [4], and this has been supported by additional prospective trials [5]. As the use of lung cancer screening increases globally, the prevalence of pulmonary nodules is likely to increase.

The main concern regarding pulmonary nodules is the possibility that they may be of malignant aetiology. There are established guidelines that stratify the likelihood of malignancy based on risk factors [6, 7]. Based on these prediction models, an investigation strategy is recommended, including serial computed tomography (CT) imaging or, in the case of higher-risk nodules, a positron emission tomography (PET) or biopsy. Obtaining tissue is the only definitive method of establishing a diagnosis, and transbronchial biopsy has been the conventional method for doing this. The sensitivity of transbronchial biopsy ranges from 14 to 63% depending on the size and location of the pulmonary nodule [8-10]. Even with fluoroscopic guidance, in combination with other sampling methods, such as washings and brushings, the yield is still highly variable [10, 11]. Radial endobronchial ultrasound (R-EBUS) with guide-sheath (EBUS-GS) was developed in the 1990s and has augmented the diagnostic yield of pulmonary nodules [12-15]. A limiting feature of transbronchial forceps biopsy (TB-FB) is the small biopsy specimens obtained. Forceps biopsies are also predisposed to crush artefacts, leading to poor preservation or alteration of cellular architecture, potentially interfering with histopathologic analysis [16-18].

These limitations have given rise to alternative bronchoscopic biopsy tools, such as cryotherapy. Cryoprobes were used initially in the 1970s as debulking tools [16]. Modifications to the technology have generated increased freezing power and enhanced the adhesive effects, allowing cryoprobes to be utilized for transbronchial cryobiopsies(TB-CB) [16]. From a diagnostic perspective, TB-CB was originally used for the diagnosis of diffuse parenchymal lung disease (DPLD) and reliably obtained larger samples with less artifact and more alveolar tissue and yielded a diagnosis in 70-80% of patients across multiple case series and several randomized trials [19]. The reassuring safety profile has led to the increasing use of cryoprobes in the investigation of SPN, and it has been hypothesized that the larger specimens may improve diagnostic yield.

In this randomised controlled trial, we sought to evaluate the use of cryoprobes compared to forceps biopsy in the evaluation of SPNs. We aimed to determine whether transbronchial cryobiopsy would produce larger specimens and improve diagnostic yield when compared to standard forceps biopsies. We also aimed to evaluate the diagnostic yield in lesions that were challenging to access with conventional forceps and to further investigate the safety profile of this technique.

## 2 METHODS

### 2.1 Participants

**Fig. (1):**
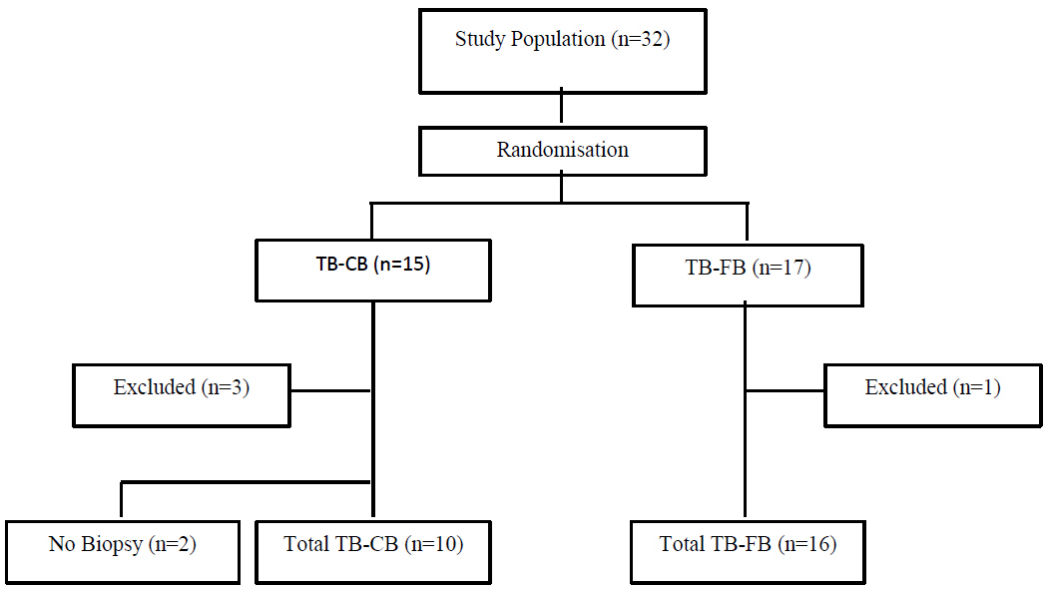
Study design. transbronchial cryobiopsy (TB-CB), transbronchial forceps biopsy (TB-FB).

This was a prospective, randomised single centre trial performed at the Royal Adelaide Hospital (RAH), Adelaide, Australia. Patients with pulmonary nodules requiring radial EBUS with guide sheath were included. Patients with endobronchial disease or mediastinal lymphadenopathy seen on the diagnostic computed tomography (CT) scan were therefore excluded. Similarly, patients with pulmonary masses above 30mm in size were not eligible. Patients who were unfit to undergo bronchoscopy and patients with bleeding or clotting disorders, including those on anticoagulants or those in whom anticoagulants could not be safely held, were excluded. Patients who were less than 18 years of age or who could not independently provide informed consent were also ineligible. Ethical approval was obtained from the Royal Adelaide Hospital Human Research Ethics Committee, and patients provided written consent. A computer-generated randomization schedule (Fig. **1**) was used to randomise patients to either conventional TB-FB or TB-CB. The nature of the procedure and requirement for general anaesthesia in the TB-CB group prevented complete blinding.

### 2.2 Intervention

All procedures were performed by respiratory registrars under the supervision of an experienced interventional pulmonologist in the Thoracic Procedure suite at the RAH. Transbronchial cryobiopsy cases were performed under general anaesthesia. Patients undergoing conventional radial EBUS with forceps biopsy either received general anaesthesia or sedation with intravenous fentanyl and midazolam.

Pulmonary lesions were mapped manually using the Kurimoto method of bronchial branch tracing from computed tomography (CT) scans of the chest with no navigational software used [20]. Under single planar fluoroscopic guidance, the radial EBUS probe (K-201/K-203, Olympus, Tokyo, Japan) was used to visualize the lesion. The EBUS probe was then removed, and the guide-sheath remained in place. Patients in the forceps group received 5 biopsy samples. In the cryobiopsy arm, the guide sheath was removed, and the 1.9mm Cryoprobe (ERBE 1.9 cryoprobe; Germany) was inserted using fluoroscopic guidance into the same subsegmental airway. A freeze time of 5 seconds was utilized based on published recommendations and our experience with diffuse parenchymal lung disease. The bronchoscope and cryoprobe were then removed en bloc. Only one cryobiopsy sample was taken. All patients received bronchial washings and brushings as per the standard of care.

All patients receiving transbronchial cryobiopsy (TB-CB) were intubated using an endotracheal tube (Rusch, Bronchoflex) with two-ports. This allows the bronchoscope and a balloon blocker catheter to be passed simultaneously. The balloon could be inflated immediately after removing the bronchoscope and cryoprobe to retrieve the specimen, thus safely tamponading any bleeding.

Any difficulty in accessing a lesion after the guide sheath was removed was considered a failed cryobiopsy procedure. In cases where the cryoprobe was initially used, the operator would revert to standard methods with forceps to re-attempt biopsy.

The primary outcome was diagnostic yield, which was defined as the number of patients in whom diagnostic samples were obtained divided by the total number of patients undergoing bronchoscopy. Samples demonstrating histopathological evidence of malignancy or a specific benign pathology, such as infection, granulomatous inflammation or benign tumours were considered diagnostic. Samples demonstrating non-specific benign diagnoses, such as inflammation proceeded to have secondary diagnostic procedures or had a minimum of 6-months of surveillance. Secondary outcomes included the diagnostic yield in subsets not easily accessible with traditional forceps, the adequacy of sampling assessed as biopsy size, and the complication rate assessed post-procedure.

### 2.3 Statistical Analysis

Continuous variables are described as mean and standard deviation or median and interquartile range as appropriate to distribution, and dichotomous variables as number and percentage. Baseline characteristics in both groups were compared with Student t-tests or Wilcoxon rank-sum tests for continuous variables as per distribution and chi-square tests for dichotomous variables. Outcomes in both groups were compared with chi-square tests. Analyses were undertaken with Stata version 13.0 (Stata Corporation), and statistical significance was set at 0.05. Results were reported based on the randomisation group.

## 3 RESULTS

### 3.1 Patient Characteristics and Recruitment

A total of 32 patients (19 males, Fig. **1**, Table **1**) were recruited between 2016-2019. Recruitment ceased in 2019 due to high demand and limited resourcing for general anaesthetic procedure lists, as well as a change in practice towards using disposable cryobiopsy probes, which interfered with the consistency of implementation of the procedural protocol. Fifteen patients were randomized to the cryotherapy (TB-CB) arm, and 17 patients were randomized to the conventional transbronchial forceps biopsy (TB-FB) arm. One patient in the TB-FB group was excluded as the lesion had resolved by the time of the procedure. Three patients in the TB-CB were excluded. Two of these patients had lesions that had resolved at the time of the procedure, and one had FDG-avid mediastinal lymphadenopathy on a PET scan, and a linear EBUS was the required diagnostic procedure. Two additional patients in the TB-CB group did not receive a cryobiopsy as, in one case, the lesion was too proximal for safe sampling, and in the second patient, the cryoprobe could not be manoeuvred into the correct airway. As a result, 28 patients total were retained for the analysis. The mean ages of participants in the cryotherapy and forceps arms were similar at 63 +/- 13 years and 64 +/- 17 years respectively (Table **1**).

### 3.2 Lesion Characteristics

Nodules in the cryobiopsy arm were, on average, 19x21 millimeters compared to 25x23 millimeters in the forceps arm (p=0.71 and 0.22 for shortest and longest dimensions, respectively; Table **1**). Median lesion volume was slightly smaller in the forceps group (360 [IQR 192-800] *versus* 459 [IQR 276-1012] mm^2^, p=0.38). Distance from the pleura was also very similar across groups, with median distances of 10mm (IQR 3-30) in the cryobiopsy group and 8mm (IQR 3-30) in the forceps group (p=0.94). 50% of participants had right upper lobe nodules, and 25% had left upper lobe nodules. The remaining 25% of nodules resided in the right lower lobe, lingula and left lower lobe.

### 3.3 Biopsy Characteristics

Biopsy sample sizes in the cryobiopsy arm were generally larger than the forceps biopsy arm, with sizes of 7 (IQR 2-22) mm and 2.5 (IQR 1-5)mm (p<0.0001), respectively. The EBUS probe demonstrated a concentric image consistent with the probe located within the lesion in 22 out of 28 patients. Conversely, an eccentric image suggesting the probe was adjacent to the lesion was seen in 4 out of 28 patients. Three of these patients were randomised to the TB-CB arm. In 2 out of 28 patients, the lesion could not be identified.

### 3.4 Diagnostic Yield

The diagnostic yield in the cryobiopsy arm was 91.7% (11 out of 12 patients). This was numerically higher than the forceps arm, which had a diagnostic yield of 68.8% (11 out of 16 patients); however, statistical significance was not achieved (p=0.14). The overall diagnostic yield for all patients was 78% (22 out of 28 patients) (Table **2**).

When the lesion was visualized with the EBUS probe, 90.9% (10 out of 11 patients) of patients in the cryobiopsy arm had a positive diagnosis (Table **2**). Whilst not statistically significant, the yield in the forceps biopsy arm when EBUS visualisation was achieved was lower at 73.3% (11/15 patients). In the two cases where the lesion could not be visualised using EBUS, a diagnosis was still made in the individual patient randomized to the cryobiopsy arm.

When a concentric radial EBUS image was achieved, the diagnostic yield in the cryobiopsy arm was 87.5% (7/8 patients). In the forceps biopsy arm, the yield was 71.4% (10/14)(p=0.61). Of the 4 patients that had eccentric radial EBUS images suggesting an adjacent lesion to the probe, the 3 patients randomised to the cryobiopsy arm had a positive diagnosis, as did the 1 patient who received a forceps biopsy.

There also appeared to be a trend towards increased diagnostic yield for cryobiopsy of lesions located within the left upper lobe (p=0.053).

Of the total 28 patients included in this study, 22 patients had a malignant diagnosis. Fifteen (53.6%) had a diagnosis of pulmonary adenocarcinoma (Table **3**). The remaining 6 (21%) patients had benign aetiologies with evidence of infection or inflammation on biopsy. The diagnostic yield for malignant lesions was numerically higher in the cryobiopsy arm at 90% (9/10) compared to the forceps biopsy arm at 66.7% (8/12), however, statistical significance was not achieved (p=0.19). There was sufficient tissue for ancillary testing and molecular testing of anaplastic lymphoma kinase (ALK) gene, ROS1 oncogene, and epidermal growth factor receptor (EGFR) in 80% (4/5) in the forceps biopsy arm and 85.7% (6/7) in the cryobiopsy arm. A statistically significant difference between the groups was not achieved (p=0.79) (Table **3**).

A subset of patients randomized to the TB-CB arm received additional TB-FB biopsies at the proceduralists discretion. Hence, a comparison of the procedural outcomes can be made in the same patient. Three patients had positive diagnoses with TB-CB but inconclusive results with transbronchial forceps biopsy. Conversely, four patients had an inconclusive or unobtained biopsy from TB-CB but had confirmation with the forceps. In two of these cases, the lesion was either too proximal for a safe biopsy or the cryoprobe could not be manoeuvred into the correct subsegmental airway. For lesions where a diagnosis was made only *via* cryobiopsy, the median nodule size on CT imaging was small at 117 (IQR 114-225) mm^2^. In comparison, there was a median size of 619 (869-7324) mm^2^ for those nodules in which a diagnosis was only achieved with forceps and not with cryoprobe.

### 3.5 Repeat Procedures and Complications

Further diagnostic procedures were required in 7 patients. Three patients with non-diagnostic TB-CB were referred for additional diagnostic tests. Two patients had CT-guided biopsies, which identified adenocarcinoma *in situ* in one patient and confirmed a true negative eosinophilic inflammatory lesion in the second. The third patient had a repeat radial EBUS, which also confirmed a true negative benign diagnosis. Of the 4 patients with negative TB-FB results, 2 were referred for CT-guided biopsy (1 lesion resolved prior to biopsy, 1 poorly differentiated SCC), and two patients underwent repeat EBUS-GS (Well differentiated neuroendocrine tumour and non-small cell lung carcinoma).

Bleeding was the most common complication, with an incidence of 28.6% in the TB-CB arm and 3.6% in the TB-FB arm. All instances of bleeding were mild and resolved with a combination of suction and iced normal saline and/or topical adrenaline. There was no major bleeding or pneumothorax.

## 4 DISCUSSION

For the last 40 years, flexible bronchoscopy has facilitated transbronchial biopsy of solitary pulmonary nodules [21]. The addition of radial EBUS to transbronchial biopsy to provide real-time visualization has been the gold standard for biopsy of pulmonary nodules. In view of its efficacy and safety, the American College of Chest Physicians (ACCP) guideline recommends R-EBUS as the preferred guiding technology for patients with suspected lung cancer (Grade IC) [9]. Despite this, the diagnostic yield of R-EBUS with fluoroscopic guidance has plateaued at approximately 70%, as investigated in multiple meta-analyses [12-15]. The limitation in yield reflects inadequate navigation to a pulmonary lesion and the lack of real-time evidence of biopsy sampling. The diagnostic yield from forceps biopsy in this trial was 68.8% (11/16), which is consistent with this pre-existing literature and also aligns with the historical diagnostic yield at the Royal Adelaide Hospital, which has typically been approximately 70%.

The limited diagnostic yield has resulted in a significant investigation into novel navigational strategies, including electromagnetic navigational bronchoscopy (ENB), virtual bronchoscopic navigation (VBN), Robotic-assisted bronchoscopy (RAB), and cone beam computed tomography (CBCT). Each of these techniques has a reported diagnostic yield ranging from 69.4% to 78.2% [22]. Similarly, significant investigation into novel sampling tools has yielded alternatives to the standard brushing and forceps approach. Transbronchial needle aspiration (TBNA) and bronchoscopic trans- parenchymal nodule access (BTPNA) are commonly used, the latter being a potential option for bronchus sign negative nodules [23-26]. The transbronchial cryobiopsy tool represents a simple diagnostic alternative that has demonstrated long-standing diagnostic efficacy in interstitial lung disease [27] and has only recently been described for lung nodule investigation.

Schumann *et al*. [28] were the first to report the use of radial EBUS with cryobiopsy for pulmonary nodules. Patients received both forceps and cryobiopsy with the order determined by randomisation. They reported an overall diagnostic yield of 60.5% (23/39 patients) and 74.2% (23/31) when the lesion was visualised by endobronchial ultrasound. EBUS visualisation is already known to correlate with diagnostic yield [29-37]. Our protocol differed from Schumann *et al*.’s as the patients received either cryobiopsy or forceps biopsy. A minority of patients (7 patients) received both biopsy methods at the proceduralists' discretion. Only TB-CB achieved a diagnosis in 4 of these patients, and only TB-FB confirmed a diagnosis in the other 3. Nasu *et al*. [38] had similar results where 7 patients were positive on forceps but negative on cryobiopsy. The authors reported difficulty advancing the cryoprobe where there were multiple airways, and there was thus a possibility that TB-CB was not obtained from the same site as TB-FB. This was the case with one patient in our study, where the probe could not be properly introduced into the correct airway. Our study noted that lesions in which only cryobiopsy was successful were markedly smaller when measured from CT imaging.

The position of the radial-EBUS probe relative to the lesion has also been demonstrated to influence diagnostic yield [35, 36]. One study suggested this was only relevant to lesions smaller than 3 centimetres [36]. Kho *et al*. studied the performance of cryobiopsy for nodules adjacent to the probe determined by eccentric radial EBUS images. A numerical increase in diagnostic yield from 22% to 66.7% was achieved, though statistical significance was not reached [39]. The rationale for the improvement is that the cryoprobe takes a sphere of frozen tissue rather than just sampling in a forward direction [28, 40]. In Kho *et al*.’s study, 57.2% of non-diagnostic forceps biopsies were bronchial epithelium, suggestive of inadequate lateral biopsy [39]. In our cohort, eccentric EBUS images from adjacent nodules were seen in 4 patients, and a diagnosis was achieved in 3/3 randomised to cryobiopsy.

Overall, systematic review and meta-analysis of the existing small numbers of retrospective [38, 39, 41, 42] and prospective trials [43-46] comparing EBUS-CB to EBUS-FB have shown a pooled diagnostic yield of 77% for cryobiopsy and 72% for forceps biopsy, respectively [47]. Our study demonstrated congruent results with similar diagnostic yields without a significant difference in diagnostic yield between transbronchial cryobiopsy and transbronchial forceps biopsy. Specifically, diagnostic yields were similar compared to those trials where EBUS-GS guidance was employed, as in our study [28, 38, 39]. There have also been recent novel ventures in peripheral pulmonary lesion diagnostics. Virtual navigation bronchoscopy has been prospectively combined with cryobiopsy in the sampling of peripheral pulmonary lesions with a reportedly similar diagnostic yield of 74% (n=37/50) [48]. Additionally, CT-transthoracic biopsy has been compared to EBUS-guided cryobiopsy in a randomised controlled multicentre study of 48 patients. Diagnostic yields were comparable between the two at 93.8% and 85%, respectively [49].

Minor bleeding is common with cryobiopsy [50]. This has been attributed to larger biopsy specimen size but may also be attributed to en-bloc removal of the cryoprobe and bronchoscope to retrieve the specimen, allowing blood to pool at the biopsy site. The standard transbronchial forceps biopsy technique allows minor bleeding to be immediately suctioned or tamponaded whilst the scope remains in place. Our study was congruent with published literature, demonstrating a higher rate of minor bleeding, requiring minimal intervention to resolve. Similar to Schumann’s feasibility study, there were no pneumothoraces that may be attributable to the use of fluorosocopy and the fact that only lesions visualised under EBUS were biopsied.

### 4.1 Study Limitations

There were several limitations to our study. The requirement of Transbronchial cryobiopsy to be performed under general anaesthesia meant that complete blinding of patients and investigators was not possible. Our cohort was also small and not powered to demonstrate superiority between tests. Longer waiting times for availability on a general anaesthetic list at our institution lead to high rates of patients declining recruitment and randomisation, which was the main contributor to the small cohort size. Due to the possibility of underlying malignancy, it was not appropriate to wait longer, and patients frequently selected the readily available alternative, which was a non-randomised, standard-of-care, transbronchial forceps biopsy under procedural sedation. Differences in anaesthetic type may have contributed to the difference in diagnostic yield; however, our yield was not dissimilar to other studies. Historically, for the proceduralist involved, the overall yield over many years for TB-FB is 70-80%. At the time of this study, the disposable cryoprobes were not yet available, and we were, therefore, limited to the non-disposable 1.9mm probe. The limitation of this probe, as also highlighted by Nasu *et al*. [38], is that when used with the diagnostic bronchoscope with a 2.0mm working channel, the EBUS-GS has to be removed, and it is not possible to be sure that the same area as found by EBUS is being sampled. With the advent of the 1.1mm disposable cryoprobe, current or future studies will be able to overcome this limitation by passing the cryoprobe directly down the 1.7mm EBUS-GS.

## CONCLUSION

In our randomised controlled trial, cryobiopsy provided larger biopsy specimens and facilitated a high diagnostic yield which was comparable to the forceps biopsy standard of care. There was no increase in the incidence of major complications. Respiratory physicians may consider cryobiopsy in the evaluation of pulmonary nodules and, in particular, for small nodules and those adjacent to the probe. Further prospective randomised trials with larger cohorts would be required to confirm our results.

## AUTHORS’ CONTRIBUTION

(I) Conception and design were proposed by Michelle Wong, Phan Nguyen

(II) Administrative support was provided by Phan Nguyen

(III) Provision of study materials or patients was provided by Phan Nguyen, Hubertus Jersmann, Mark Holmes, and Michelle Wong.

(IV) Collection and assembly of data were done by Michael Brown, Michelle Wong, and Phan Nguyen

(V) Data analysis and interpretation were performed by Phan Nguyen, Michael Brown, and Michelle Wong

(VI) Manuscript writing was done by all authors.

(VII) Final approval of the manuscript was given by all authors,

## Figures and Tables

**Table 1:** Patient demographics and nodule characteristics.

-	**Forceps Biopsy**	**Cryobiopsy**	**p-value**
**Patients**	-	-	-
Age	64 +/- 17	63 +/- 13	1.00
Gender	-	-	-
Male	10 (58.8)	9 (60.0)	0.95
Female	7 (41.2)	6 (40.0)	-
**Lesion**	-	-	-
Size	25x23	19x21	-
Area (mm^2^)	459 (275-1012)	360 (192-800)	0.38
Distance from pleura (mm)	8 (3-30)	10 (3-30)	0.94

**Table 2:** Diagnostic yield overall and by subgroup. EBUS (Endobronchial ultrasound).

-	**Forceps Biopsy % (n)**	**Cryobiopsy % (n)**	**p- value**
**Biopsy Method**	68.8 (11/16)	91.7 (11/12)	0.14
-	-	-	-
**EBUS visualisation of lesion**	-	-	-
Positive	73.3 (11/15)	90.9 (10/11)	0.26
Negative	0 (0/1)	100 (1/1)	1.00
-	-	-	-
**Probe Position**	-	-	-
Within Lesion	71.4 (10/14)	87.5 (7/8)	0.61
Adjacent to Lesion	100 (1/1)	100 (3/3)	-
-	-	-	-
**Diagnoses**	-	-	-
Malignant	66.7 (8/12)	90.0 (9/10)	0.19
Benign	75.0 (3/4)	100 (2/2)	0.44

**Table 3:** Malignant diagnosis histological breakdown and sufficiency for ancillary testing by subgroup. Non-small cell lung carcinoma (NSCLC), anaplastic lymphoma kinase (ALK) gene, ROS1 oncogene, epidermal growth factor receptor (EGFR).

**Malignant Histological Diagnosis**	**Forceps Biopsy % (n)**	**Cryobiopsy % (n)**	**p- value**
NSCLC - adenocarcinoma	58.3 (7/12)	80.0 (8/10)	0.28
Sufficiency for ancillary testing (EGFR/ALK/ROS1)	80.0 (4/5)	85.7 (6/7)	0.79
NSCLC – Squamous cell carcinoma	25.0 (3/12)	10.0 (1/10)	0.36
Colorectal Carcinoma	8.3 (1/12)	10.0 (1/10)	0.89
Well differentiated neuro-endocrine tumour	8.3 (1/12)	0 (0/10)	-

## LIST OF ABBREVIATIONS

RAH: Royal Adelaide Hospital
CT: Computed tomography
TB-CB: Transbronchial cryobiopsy
TB-FB: Transbronchial forceps biopsy
ALK: Anaplastic lymphoma kinase
EGFR: Epidermal growth factor receptor
